# Preventive Treatment

**Published:** 1882-04

**Authors:** C. A. Marvin

**Affiliations:** Brooklyn, N. Y.


### ARTICLE V.

### *Preventive Treatment.*

BY C. A. MARVIN, D. D. S., BROOKLYN, N. Y.

[Extract from a paper read before the New York Odontological Society.]

Purely preventive treatment must begin far back, antedating birth, conception, marriage. In the girlhood of the yet future mother the instruction should be given, which, if followed, will secure uniform physical development, perfect nervous balance, a healthy circulation, good digestion —in a word, robust health. This is the time for, and this *is* preventive treatment. It consists of nutritious diet, regularity of habits, exercise in the open air—such exercise as employs all the machinery of the human frame, as walking, horseback-riding, rowing; a style of dress which does not hinder the free action of the internal organs, which does not distort the body or weigh unduly upon the abdomen; nor overclothe one part, leaving another unprotected; regular and consistent habits of thought; the cultivation of equability of temper; sufficient sleep at the proper hours for sleep.

Such habits of life, many of which I know are unfashionable, will prepare a woman to transmit to the children she may bring into the world an inheritance of health of incalculable value and permanent duration.

But not only do the future mothers of children need such instruction; young men need it also. Excesses in youth plant seeds of weakness and disease which bear terrible fruit in mature manhood, and are bequeathed by fathers to children with sad certainty. Were it not that evidences of the unwelcome inheritance are seen in the organs which come into our hands for treatment, this particular thought need not have been introduced in a dental paper. The fact existing—cases illustrating and proving its truth coming under the eye of dentists freely—its introduction in this paper is eminently proper.

Much of this purely preventive service may be done by the dentist if he is true to his high calling and alive to the interests of his race. Wise suggestions to mothers of young daughters, to young women themselves, will accomplish much. Great judgment will be necessary in doing this kind of work, that delicacy be not offended and the dentist called an intermeddler. No man has a right to offend the delicacy of any woman, nor does the powerful shield of professional privilege protect from just censure the man who assumes such a right.

Physicians may be and often are obliged to do what is very trying to the delicacy of their patients, but when done under the stress of necessity and with propriety of demeanor, no offence is taken. Nor should there be. So dentists can perform such extremely delicate service as has been indicated, and have it kindly, gratefully received. It is a kind of service almost wholly neglected. Few dentists are qualified to do it, and fewer still are courageous enough to undertake it.

Now it may be said that such advice to children, especially to girls, should come from parents, or if from any other, from the family physician. If parents will do it, well; but, as a rule, they are not informed on such subjects. If their attention is called to the bad condition of their children's teeth, they simply remark, "Yes, my children inherit bad teeth from me," without a thought of the possibility of preventing a recurrence of the evil in the next generation.

If it is conceded that the physician may with propriety so instruct young people, then may the dentist, and I maintain that the family dentist should sustain as confidential relations to his patients in things pertaining to his specialty, as does the physician in his general practice. If this doctrine were generally accepted (happily it is to some extent) by the profession and the public, a new and mighty influence for higher attainment would at once be put in operation.

*All dentists should be qualified* to sustain such relations to their patients, and to perform such service. Just here a searching question might be put: What proportion of the practicing dentists of to-day are so qualified?

As this question is pondered, the necessity of a more thorough education of students of the useful and arduous and exacting profession of dentistry is clearly seen; for it will be at once admitted that we can never have a profession equal to the high demands upon it till such an education is insisted on; till well-rounded men issue from the educational institutions of dentistry; till diplomas mean what they are supposed to mean; till college degrees invariably speak the truth, and they who own them shall have earned them, and can justly be proud of them, and are entitled to the honor they are intended to confer, and can take an equal place among the learned in other professions and among the scientific and thoroughly furnished men of their generation. To avoid misinterpretation, let me repeat what was stated at an earlier stage of this discussion, that if these remarks are taken to mean what men of this high class are totally wanting, such an inference is beyond the intention of this paper. There are some such men but we need more.

If a student *will*, he may acquire a good foundation of instruction in dental schools. But suppose he is indifferent —is he put to such a test as a thoroughly-furnished mind only can endure? And does he find that indifference to study or a mistaken estimate of his ability insures failure? Are the hundreds who are let loose upon the community from our dental colleges, year by year, competent men? Are they men who are capable of adorning the profession? Are they men of culture, men of thought, men who appreciate the value of discoveries which their predecessors have made, and able in many things to begin where *they* stopped, and thus making each generation an advancing one in the line of true progress? If so, well and good. If not, they ought to be, and should be required to be. Is this smilingly dubbed an Utopian idea? Then let us who are convinced of its soundness pray and labor that our land may speedily become Utopia, and our age an Utopian age! Let us exercise ourselves that one of these ends shall be realized, viz., the more thorough equipment of practitioners of our specialty. Not an equipment of glittering instruments, the furniture, and costly surroundings; these money can buy and an ignoramus can own ; but a mental equipment, well chosen and complete,—this much is not unattainable. It may possibly reduce the number of graduates; it will quite likely diminish the number of students. For a higher and severer test at the end of a college course will necessitate a better foundation for general knowledge at the beginning; and they who are unable to express their thoughts in grammatical language, or to make a proper choice of letters in spelling the words they wish to use, will hardly feel encouraged to apply for admission to a college to pursue a course of professional study. Just in this respect there is need of reform, for it is absurd to see young men received to a course of scientific instruction whose common education is not up to the standard of a district school. And yet this is seen in some of our dental colleges. Now, this can and should be corrected.

When this end (the higher education of dental students) is attained, the other (the instruction of patients) is sure to follow in its time. For a man who is thoroughly informed himself, when he sees the want of the information he can give, cannot refrain from giving it. He must speak. Silence is intolerable, for he deems it ungenerous. Good suggestions will flow out of such a man's mouth almost involuntarily, clothed with such an air of sincerity and in words of such earnestness as will command attention and work good.

